# Safety and Effectiveness of a Novel Color Corrector Serum for Causing Temporary Changes to Tooth Shade: A Randomized Controlled Clinical Study

**DOI:** 10.3390/dj12070197

**Published:** 2024-06-27

**Authors:** Mauro Pascolutti, Alex Tomic, Kimberly R. Milleman, Jeffery L. Milleman, Laurence J. Walsh

**Affiliations:** 1 Hismile, Burleigh, QLD 4220, Australia; alex@hismiletteth.com; 2 Salus Research Inc., Fort Wayne, IN 46825, USA; kmilleman@salusresearch.us (K.R.M.); milleman@salusresearch.us (J.L.M.); 3 School of Dentistry, The University of Queensland, Herston, QLD 4006, Australia; l.walsh@uq.edu.au

**Keywords:** tooth shade, color change, whitening, shade measurement, cosmetics

## Abstract

Tooth color is a major driver of facial esthetics. While permanent changes in tooth shade can be achieved by bleaching and restorations, there is a need for cosmetic products that can cause reversible color changes. This randomized controlled clinical study assessed the effectiveness and safety of a novel color-correcting product (Hismile™ V34 Color Corrector Serum™) versus a placebo (vehicle control lacking the color-change dyes). A single-center, randomized, controlled, examiner-blind, two-group, parallel design, single-use study design was followed. The test products were applied on a cotton bud for 30 s, and then, rinsed off. Tooth shade for maxillary central incisors was measured at baseline, immediately, and at 30 and 60 min, using the Vita Bleachedguide 3D-Master^®^ Shade Guide and the EasyShade Advanced 4.0 spectrophotometer (for determining values of L*a*b*). The subjects (N = 60) had a baseline shade of 1M2 (rank 9) or darker. A single application of the test product resulted in an immediate and significant (*p* < 0.001) three shade improvement (26.2%) according to the shade guide, and the same significant benefits extended to 30 and 60 min. The placebo product did not alter tooth shade (*p* = 0.326). These changes were accompanied by significant improvements in the L value (whiteness) up to 30 min, and a reduction in b* (yellowness) for up to 60 min. Two-thirds of subjects using the test product stated in a survey that their teeth appeared both whiter and brighter. No safety issues arose from the use of the test product or vehicle control. These results indicate that using a color corrector can achieve worthwhile changes to tooth shade for up to 60 min.

## 1. Introduction

Tooth color plays a major role in how individuals assess others for attractiveness and vitality [1,2,3,4]. Even relatively minor changes in the lightness and yellowness elements of tooth color can influence attractiveness [4]. Those with teeth that are more yellow and darker tend to be rated as older and less attractive. The size of this effect of tooth shade appears to have increased over the past decade, since older studies did not report such strong connections compared to more recent investigations [5,6,7]. This can be explained by the greater awareness of individuals regarding tooth shade because of the use of smartphones and social media [8]. 

For causing permanent changes to tooth shade, the use of veneers [9] and vital bleaching techniques [10,11] are well-established methods. On the other hand, tooth shade can also be altered on a temporary basis by applying dyes that enhance the reflection of short wavelengths of visible light from teeth, such as violet and blue. This should make the teeth appear brighter and also less yellow [12]. This concept has been described in the patent literature since 2006. The first attempt involved adding a commercial violet food dye, containing red 3 dye and blue 1 dye to regular toothpaste, to enhance the color of teeth [12]. A later concept was adding blue covarine dye (CI 74160) to toothpaste [13,14,15,16]. While the latter approach has been used in several commercial toothpastes in Europe and Australia, blue covarine dye is not a permitted additive in the USA, and its poor solubility in water limits its application in toothpaste products in other global markets. 

An alternative cosmetic concept has been to apply highly water-soluble non-toxic food coloring dyes in a concentrated form for temporary color correction of teeth. The chosen dyes should be color-stable in terms of the range of intra-oral pH values, so that the coloring effect is predictable. V34 Colour Corrector Serum™ (V34CC) (Hismile, Burleigh, QLD, Australia) is a viscous hydrophilic glycerin-based liquid containing high concentrations of D&C red 33 and brilliant blue FCF. It is designed to give a temporary color-boosting effect that lasts for as long as the two dyes remain bound to pellicle. 

Since no past studies have explored the clinical performance of a two-dye color-change product for short-term direct application to teeth, the aim of the present study was to assess the color change caused by a single application of V34CC or a placebo lacking the dyes, and to assess two relevant clinical safety aspects of this cosmetic agent, namely, gingival irritation and tooth hypersensitivity. 

## 2. Materials and Methods

Efficacy for colour change was assessed by using the well-established colorimetric and visual assessments [14,17] that are employed in clinical practice as well as in laboratory and clinical studies of novel cosmetic approaches. The shade changes affecting the maxillary central incisor teeth were assessed using the Vita Bleachedguide 3D-Master^®^ shade guide (VITA Zahnfabrik, Bad Säckingen, Germany) and the VITA Easy Shade Advanced 4.0 digital spectrophotometer (to measure L*a*b* values) across three time points (immediate, 30 min and 60 min). Safety was assessed through oral examination of soft and hard tissues, and examiner ratings of irritation, as well as by using a questionnaire. The latter tracked self-reported tooth hypersensitivity, as well as overall satisfaction for enhancing tooth shade. The null hypothesis was that there would be no difference between the test and placebo products.

### 2.1. Study Design

The study used a randomized, single-center, examiner-blinded, parallel design. Subjects were randomized 1:1 between the test product (Hismile^TM^ V34 Colour Corrector™ Serum, Hismile, Burleigh, QLD, Australia) and a placebo vehicle control that was identical but lacked the color-change dyes, based on a computer-generated randomization schedule prepared in advance of the study. Randomization was undertaken by an independent staff member who was blinded to the treatment protocol. The study was conducted at Salus Research, Inc (Fort Wayne, IN, USA), during January 2024, in accordance with proposed guidelines for current good clinical practices (cGCPs), and in compliance with the United States Federal Regulations governing informed consent (21 CFR 50), Institutional Review Boards (21 CFR 56), clinical investigations, and applicable regulations governing sponsor and investigator conduct (21 CFR 312/812).

The test product (V34CC) contained two water-soluble dyes (CI 17200/D&C Red No. 33, Cl 42090 FD&C Blue No. 1) in a vehicle of glycerin, water, flavors (sorbitol, xylitol, sucralose, peppermint oil), thickeners (cellulose gum, hydrated silica), emulsifiers (polysorbate 80), tetrasodium pyrophosphate, and preservatives (phenoxyethanol and ethylhexylglycerin). The placebo was identical in composition, but lacked the two dyes.

**Figure 1:**
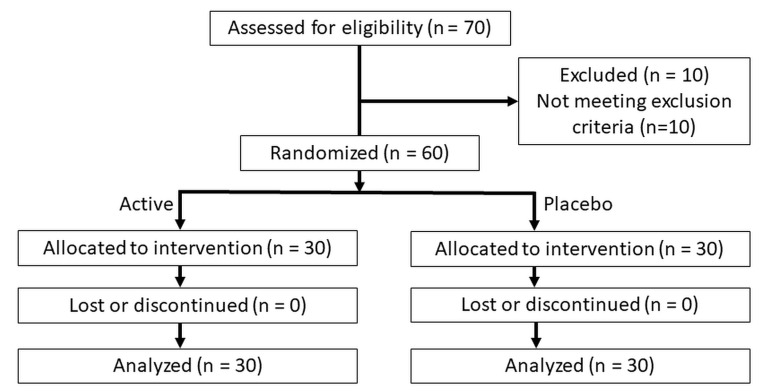
CONSORT diagram.

**Figure 2:**
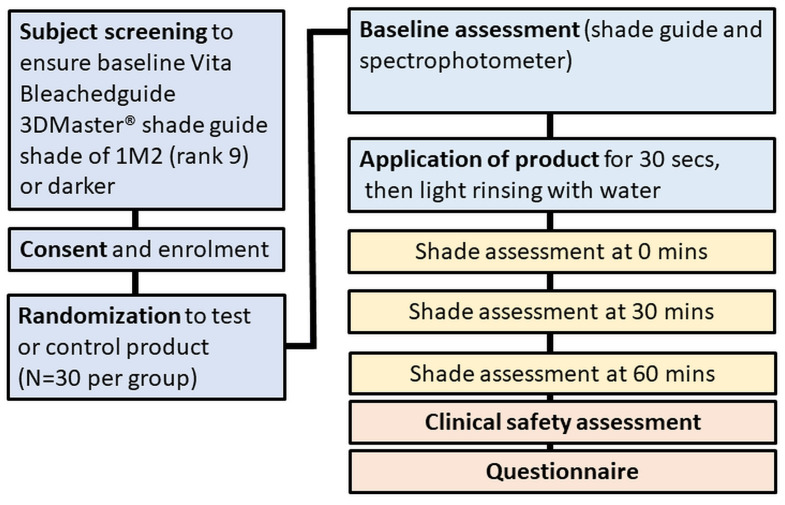
Study design.

The study was conducted according to the guidelines of the Declaration of Helsinki, and received human research ethics approval (WHT2024.01) from the U.S. Investigational Review Board Inc, (Miami, FL, USA) which is a US Department of Health and Human Services (DHHS)-registered independent ethics committee (#U.S.IRB2023SRI/10). Clinical trial registration was not sought as the test product is a cosmetic agent rather than a medical device, and does not make therapeutic claims, and hence, falls outside medical device regulatory schemes. The CONSORT diagram is shown in Figure 1 and the study design in Figure 2.

### 2.2. Recruitment and Selection

A total of 60 subjects, aged between 18 and 50 years of age, were enrolled. All subjects resided in Fort Wayne, IN, USA. The sample size was based on a power analysis (StatMate, GraphPad, San Diego, CA, USA). With 30 subjects per group and a repeated measures design following each subject over time, there is 95% power to detect a 1.05 Vita shade mean difference between the test and placebo for a two-sided paired *t*-test. 

Informed consent was obtained from each subject prior to participation, in line with US Food and Drug Administration Good Clinical Practice (GCP) guidelines for clinical studies. Information was given in both oral and written form, and subjects were given ample opportunity to inquire about details of the study prior to signing and dating the consent form. A copy of the signed consent form was given to each subject. 

The inclusion criteria included having good general health (based on a medical history review), good oral health, no dental prophylaxis within the last 2 months, and a minimum of 16 natural teeth with at least two maxillary incisors with a Vita Bleachedguide 3D-Master^®^ tooth shade of 1M2 (rank 9) or darker. The exclusion criteria included previous use of home whitening/tooth bleaching products or systems within six months prior to screening and a prior history of professional tooth bleaching within 1 year; participation in any study involving oral care products, concurrently or within the 30 days of screening, or participation in a whitening study within 6 months of screening; a history of allergies, adverse effects, or oral soft tissue sensitivity to any ingredients disclosed on the label; significant oral soft tissue pathology based on a visual examination; any disease or conditions, including dry mouth, which could be expected to interfere with examination or procedures used in the study; self-reported serious medical conditions; a requirement for antibiotic pre-medication prior to dental procedures; severe periodontal disease or active treatment for periodontal disease; having five or more untreated carious lesions; the presence of orthodontic appliances, peri/oral piercings or removable partial dentures; significant oral soft tissue pathology; a history of enamel fluorosis, fixed orthodontic appliance in the maxillary anterior region, or caries or restorations on the anterior teeth; and the presence of generalized tooth recession, malocclusion, or overlapping teeth in the anterior region.

The date of the last dental visit when a prophylaxis was performed, and the frequency of drinking coffee, tea, red wine, and dark soda drinks was noted, as well as smoking habits. For at least two hours prior to the clinical assessments, subjects refrained from toothbrushing, smoking, eating, or drinking coffee, tea, or other stain producing beverages. In addition, subjects were required to refrain from using a lipstick product to avoid interference with the examiners’ assessments of tooth shade. Tooth sensitivity at baseline was rated by subjects on a 4-point scale as absent, mild, moderate, or severe

Following the baseline examination, the test products were applied according to the treatment regimen, under supervision of study staff. Subjects remained at the clinical site until they had completed the 1 h post treatment evaluations, and during this time they did not smoke, and were not allowed to eat or drink. This ensured that no interference from colored materials from diet or lifestyle habits occurred with the shade assessments.

### 2.3. Product Application

The test or placebo products were supplied in coded packages. The materials were applied by the supervising research site team member using a cotton-tipped applicator, onto which two pumps of the material had been dispensed. The applicator was then gently moved across the labial surface of the two maxillary central incisor teeth for a total of 30 s. This was followed by gentle rinsing with the water spray from an air/water triple syringe.

### 2.4. Shade Assessments

One trained dental examiner performed all shade examinations using the Vita Bleachedguide 3D-Master^®^ tooth shade system (BSG, Vita Zahnfabrik, Bad Sackingen, Germany) and the Vita EasyShade Advanced 4.0 spectrophotometer. The shades of the maxillary central incisor teeth were assessed at the start of the visit prior to use of the products to provide a baseline, and again immediately after product use, and at 30 min and 60 min. This examiner was blinded to product usage. Maxillary incisor teeth were chosen due to their importance and prominence within the esthetic zone, and because they have a large and flat facial surface which is well suited to shade measurements using a spectrophotometer.

The single examiner performed the visual tooth color assessments under standardized lighting conditions. The lighting in the room consisted of color-corrected lighting in the range of 5000 kelvin. A blue bib was placed over the subject’s clothing. As mentioned previously, subjects were not permitted to wear lipstick. All assessments were performed in the same room. In addition, outside light was controlled by covering the windows. Tooth shade was scored on the selected maxillary central incisors using the Vita Bleachedguide 3D-Master^®^ shade guide, in line with the manufacturer’s instructions. The tab marking system included interpolated shade guide units (sgu), ranging from 1 to 29 sgu. The lightest tab, 0M1, is assigned a rank of 1, and the darkest tab, 5M3, is assigned a rank of 29. Subjects’ shade-rank scores were determined at each time period by calculating the mean across the two maxillary central incisors.

Objective measurements of tooth color were undertaken using the Vita EasyShade Advanced 4.0 spectrophotometer (VES). This measures tooth color based on the CIELAB color notation system. L* denotes lightness, while a* and b* denote green–red and blue–yellow coordinates, respectively. Operation of the spectrophotometer followed the manufacturer’s instructions, which included calibration (automated and manual). The VES was used to measure a single tooth area (middle third) on each of the two maxillary central anterior incisors. The probe tip was placed perpendicular and flush to the tooth surface. Data were recorded for the L*C*h*a*b* color coordinates in the CIEL*a*b color space for the measured area of the tooth.

### 2.5. Safety Assessments

A secondary objective of the present study was to determine the safety of the test and placebo products, based on gingival irritation and tooth hypersensitivity. Before any product use, each subject made a self-assessment of tooth sensitivity using the visual analog scale (VAS) rating scale on the provided form, by placing a mark anywhere between the demarcations for 0 and 10 to indicate the level of perceived sensitivity. Immediately after product use, subjects made another self-assessment of oral irritation using the VAS rating scale on the provided form. 

The presence of oral mucosal reactions to the test products was assessed by clinical examination. The teeth and oral soft tissues (lips, buccal, labial and sublingual mucosae, tongue, hard and soft palate, uvula and oropharynx) were examined for signs of reddening or /inflammation, ulceration, white patches, and desquamation or sloughing of mucosal tissues, and the findings were recorded. 

Clinical research personnel asked subjects about the occurrence of any adverse events. All observed or volunteered adverse events, regardless of treatment group or suspected causal relationship to the study product, were recorded. Subjects were also questioned verbally regarding gingival irritation in their exit questionnaire.

The oral soft tissue findings were tabulated and summarized by treatment group for all exams. The number and percentage of subjects experiencing adverse events was tabulated by treatment using a standard coding dictionary. Adverse events were summarized according to their relationship to the product used and according to severity.

### 2.6. Questionnaire

A written questionnaire was completed by all subjects, by themselves, after the immediate post-application shade assessment. This was not a validated questionnaire. Subjects were not aware of their numerical results for immediate shade change, or of their group allocation, when completing the questionnaire. 

The questionnaire related to the two exploratory endpoints of the study. The first of these was the overall satisfaction of subjects. The three questions asked whether the test product (1) whitened the teeth, (2) brightened the teeth, or (3) concealed yellow tooth stains, immediately after application. The possible responses for each question were either agree, unsure, or disagree. 

The second element was subjects’ self-reported side effects immediately after application, such as gingival irritation and tooth sensitivity. Tooth sensitivity was rated by subjects using the visual analog scale. 

### 2.7. Data Analysis

The primary endpoint for the study was the change from baseline in tooth shade (based on the Vita Bleachedguide 3D-Master^®^ shade guide) immediately after a single use, with secondary endpoints for changes from baseline to 30 min and to 60 min. The additional secondary endpoints for the study were the changes from baseline in tooth shade immediately and after 30 min and 60 min when assessed using the VES spectrophotometer, for values of L*a*b*, and the examiner assessment (safety profile) based on a review of oral soft and hard tissues after each time point.

Parametric statistical tests (analysis of variance) were used for shade guide units and L*a*b* data as continuous variables, as data sets met the required assumptions, including having normal distributions and comparable variances. Changes in shade and percentage change from baseline were calculated. Means for the groups were compared using adjustment for multiple comparisons where appropriate. The significance of comparisons was assessed against a two-sided significance level of 0.05. Continuous variables for demographic data were evaluated using an unpaired (two-sample) t-test, while data for categorical variables were assessed using Fisher’s exact test. Statistical analyses were performed using SAS version 9.4 for Windows (SAS Institute, Sydney, Australia).

## 3. Results

### 3.1. Participants

After applying the selection criteria, a total of 60 subjects (43 females, 17 males) were enrolled. When allocated, this gave 30 in each group. All subjects completed the study, with no withdrawals, dropouts, or variations in the study protocol occurring. 

There were no statistically significant differences between the test and placebo groups for any demographic variables (age, gender, race, and ethnicity), or for baseline shade. The key parameters at baseline for the test and control groups were as follows: mean age and age range (39.7 years (range 19–50), and 37.6 years (range 18–50)); baseline smoking habits (10% and 13%); baseline Vita shade (11.7 and 12.4); baseline L (77.0 and 77.84); and baseline b* (13.9 and 14.0). 

### 3.2. Shade Changes—Vita Shades

**Figure 3:**
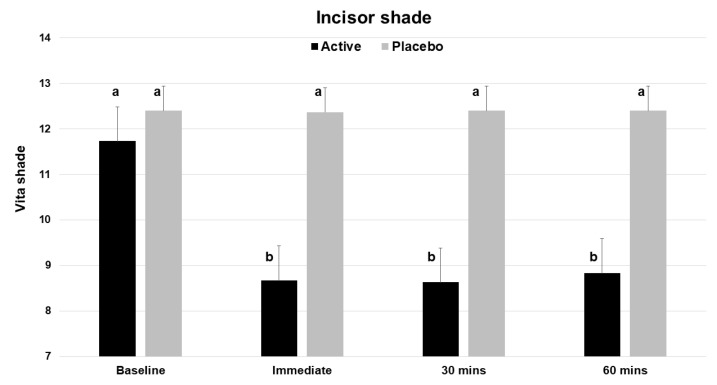
Vita Bleachedguide 3D Master shades for maxillary central incisors across the 4 time points of the study, showing the improvement (lower shade guide unit score) for the active product (V34CC), but not the placebo. This shade guide has a range of 1 to 29. Bars show mean values and standard errors. The letter a shows groups that are not different from the baseline. Groups with the letter b are significantly different from the baseline (*p* < 0.001), but not different from one another.

Based on the Vita Bleachedguide 3D Master shade guide, application of a single dose of the test product (V34CC) resulted in an immediate improvement by three shades (a change of 26.2%), which was statistically significant (*p* < 0.001) (Table 1). At the same time point, the placebo product did not alter or improve tooth shade (*p* = 0.326). The measured extent of shade change for the active product was a mean of 3.07 shade guide units (range 1 to 8), which was significantly different from the baseline, while the placebo did not cause a significant change (mean 0.03, range 0 to 1). The beneficial shade changes with the test product extended to both the 30 and 60 min time points, with each showing a significant change from baseline (*p* < 0.001) (Figure 3).

### 3.3. Shade Changes by L*a*b*

**Figure 4:**
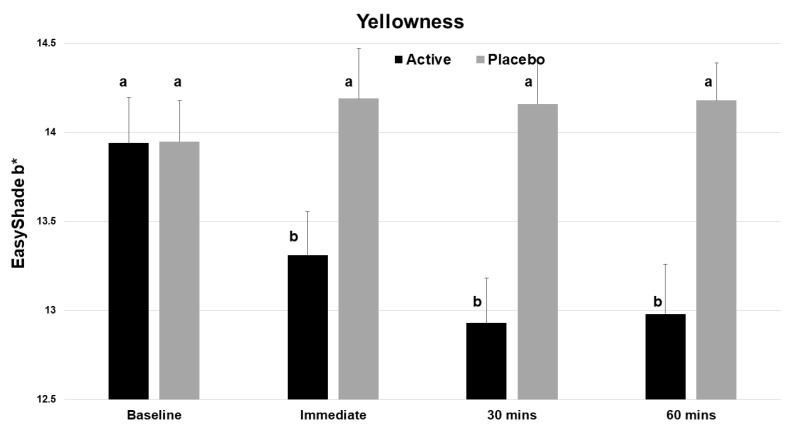
Vita EasyShade Advanced 4.0 spectrophotometer (VES) readings for b* (yellowness), showing a reduction in yellowness across the 4 time points for the active product (V34CC) but not the placebo. Bars show mean values and standard errors. The letter a shows groups that are not different from the baseline. Groups with the letter b are significantly different from the baseline (*p* < 0.001) but not different from one another.

The test product gave statistically significant improvements in the L value (whiteness) at time zero and at 30 min (*p* < 0.001, Table 1). There was a statistically significant decrease in the yellowness (b*) of the incisor teeth immediately after application (Figure 4), and this benefit continued to both the 30 and 60 min evaluations. No significant change was seen with the placebo.

### 3.4. Safety

One test subject (out of 30) reported mild tooth sensitivity following the application of the test product on their incisor teeth, but it resolved quickly (within minutes) and without any lasting sequelae. The examiner did not record any oral soft tissue adverse events following a thorough evaluation of the oral cavity at each time point for that subject, nor any others. There were no episodes reported for gingival irritation or discomfort on the Sensitivity and Irritation Questionnaire that was completed immediately after product application in both groups.

### 3.5. Responder Analysis (Subject Level)

Based on the primary outcome of the study, a responder was defined as an individual with a 2-unit or more immediate improvement in the Vita shade. When a responder analysis was undertaken, at all three time points after application, there was a significant difference in responders between the test and placebo groups (*p* < 0.001, Fisher’s exact test). All 27 responders in the study were in the active product (V34CC) group, with none in the placebo group. Overall, responders across the three time points had on average a 3-unit change in Vita shade. The minimum and maximum Vita shade changes seen in the active products group were one shade and eight shades at each of the three time points. In total, 3 of the 30 subjects in the active group had a shade change of less than 2 units immediately after application, and this dropped to 1 subject at 30 min and 60 min.

To explore whether age was a confounder in responses, analyses of Vita shades and EasyShade data parameters versus subject age were undertaken. None of these showed a statistical relationship between age and the measures of tooth shade, and responses to the test products.

### 3.6. Questionnaire

Two-thirds of the subjects (20 out of 30) using the active product stated in the written questionnaire that they believed that their teeth were whiter, while around two-thirds (21 out of 30) also indicated their teeth were brighter, and two-thirds indicated the product masked yellow stains on teeth. In contrast, in the placebo group, responses on all three questions were equivocal (47% agreement for visible whitening, 53% for visible brightening, and 47% for masking yellow stains). 

## 4. Discussion

The purpose of the present controlled clinical study was to assess the influence of a cosmetic product containing two food dyes in a glycerin vehicle for enhancing the appearance of teeth over the short term. The results show that the active product, V34CC, improved the tooth shade by on average 3 units immediately after use, and this improvement was maintained over the following 60 min. The overall response rate for a 2-unit or more immediate improvement in the Vita shade was 29/30 (96.7%). The measured extent of shade change for the active product was a mean of 3.07 shade guide units (range 1 to 8), which was significantly different from the baseline, while the placebo did not cause a significant change (mean 0.03, range 0 to 1). In all performance evaluations, the active product was superior to the placebo. Hence, the null hypothesis was not accepted.

Supporting the results from the shade assessments, objective shade analyses using data from the Vita EasyShade revealed that the test product increased whiteness (a positive change in the L axis) beyond 30 min and lowered yellowness (a negative change in the b* axis) up to 60 min. The placebo did not influence either of these parameters. 

A majority (approximately two-thirds) of the subjects in the active group of the study self-rated the appearance of their maxillary incisor teeth as being improved immediately after the application of the active product, believing them to be whiter and brighter, and that existing yellow stains were masked. On the other hand, there was no consistent view of any improvement with those who received the placebo. While the primary outcome of the study was objective changes in tooth color, it is noteworthy that measured changes in tooth shade using the VES with an experienced examiner do not correspond exactly to the subjective impression of individual participants regarding changes that occurred. The fact that some subjects in the placebo group believed there was a positive result emphasizes that subjective perception may not reflect objective outcomes for the ordinary consumer. 

The present study adds to the literature regarding the potential benefits from color-changing dyes in terms of enhancing the appearance of teeth. Unlike other work, where such dyes were incorporated into dentifrices [12,13,14,15,16], the present study used a dye mixture applied directly to the teeth. The test product contained two dyes with high water solubility (CI 17200/D&C Red No. 33, Cl 42090 FD&C Blue No. 1), giving a violet–purple color. Uptake of this dye mixture into the salivary pellicle enhances the reflection of violet light [12], which then causes the change in tooth color. Other components of the mixture (glycerin, water, sorbitol, xylitol, sucralose, peppermint oil, cellulose gum, hydrated silica, poly-sorbate 80, tetrasodium pyrophosphate, and preservatives) are not colored. This is why the placebo, which lacked the two dyes, did not cause a significant color change. 

The influence of previous consumption of foods or drinks on the salivary pellicle [17] and on the binding of the dyes must also be considered. In the present study, subjects were not exposed to agents that could potentially cause extrinsic staining. Future work should address the impact of the color-changing dye approach in situations where agents that cause extrinsic staining, such as tea and coffee, are being consumed. 

Examination of the oral cavity at baseline and after product use showed normal findings for the oral hard and soft tissues, and did not reveal any safety issues. One subject who was treated with the active product initially reported mild tooth sensitivity immediately after product use, but in their follow-up questionnaire they did not report any sensitivity at later time points. Taken together, these findings indicate that the topical use of a color-correcting dye product can give worthwhile short-term changes to the appearance of teeth, over the timeframe of 1 h used in this investigation. 

The test and placebo groups in the study were well matched for baseline shade, and for age, gender, race, and ethnicity. The study was able to assess the effect of the dye product using conventional shade measurement as well as objectively using the VES spectrophotometer. This is considered the best objective means to assess tooth shade in the recent literature [18,19,20,21,22]. Future studies should consider following a cross-over design with a suitable wash-out period so that the effect of the dye mixture and the placebo can be compared in the same individual, both in terms of objectives measures as well as in terms of perceived changes in tooth appearance. As well, comparisons could be made over time for shade changes not just for treated teeth but also for untreated teeth. 

The present study also recorded patient satisfaction and tooth sensitivity as minor exploratory outcomes. When completing the written questionnaire, subjects were not aware of their numerical results for immediate shade change, or whether they had received the active product or the placebo. Since thresholds for patient responses to changes in tooth shade can vary widely [18], it is not surprising that even when an objective measurement showed that a change had occurred from the active product, a minority did not notice this. On the other hand, around half of those in the placebo group thought that a change had occurred, when objective measurements showed no change. The “Hawthorne effect” [23,24,25,26] is present in clinical studies of dental esthetics. Test subjects may be hyper-aware of perceived improvements and report these, even when there is no objective difference. This is an extremely frustrating issue in the evaluation of dentin hypersensitivity as well [27]. These discrepancies emphasize the need to include a placebo arm in studies, to control for such effects. 

A further limitation of the present study was that only a single application of the products was performed on one day. Repeated application on the same day may alter the effect achieved. As well, the follow-up period was short. Hence, it is not known how much time must elapse for the effects on tooth shade to disappear completely. Longer studies should explore how much time is required for the influence of the dye mixture to disappear completely. This information would inform what re-application protocols may be suitable. The fact that changes in yellowness persisted for longer than changes in whiteness could reflect more subtle optical effects as bound dye is released slowly from the surfaces of teeth. 

A further point is that future studies should rate any responses of sensitive cervical dentin to the color-correcting dye using quantitative measures [28]. Only one subject noted a transient response. Nevertheless, the impact of the dye mixture on teeth with existing hypersensitive dentin needs to be assessed in future investigations.

In the present study, the treated teeth were all sound and unrestored. Further work is therefore needed on how dye mixtures can bind to the various types of tooth-colored restorative materials that may predictably be found in the esthetic zone [29], to influence their color. The influence of the overlying salivary pellicle and thin layers of dental plaque biofilm must also be considered, since these will be the major targets onto which dyes will bind. Changes in salivary film flow [30,31] may affect the color-change effect seen in patients with salivary gland hypofunction. Slower rate of clearance of the dyes may give a longer duration of effect, but this remains to be demonstrated. 

Finally, future work should also consider the performance of different dyes for short-term color correction, and compare those to dyes such as blue covarine that are already used in toothpastes in some global markets. While a range of purple dyes exist, issues with color instability with varying pH levels, poor water solubility, lack of regulatory approval, and toxicity can all impact how such dyes can be used in practical ways. 

## 5. Conclusions

The present clinical study shows that a dye mixture can cause meaningful immediate changes in the appearance of the maxillary incisor teeth, with increased whiteness and reduced yellowness over one hour when measured using objective means. No significant safety issues arose from the use of the novel product. These results indicate that a color-correction dye may be a useful cosmetic agent for achieving temporary changes to tooth shade.

## Figures and Tables

**Table 1:** Improvements from baseline shades.

	Active (V34CC)	Placebo
Vita shade T = 0 min	3.07 (*p* < 0.001)	0.03 (NS)
Vita shade T = 30 min	3.10 (*p* < 0.001)	0.0 (NS)
Vita shade T = 60 min	2.90 (*p* < 0.001)	0.0 (NS)
L (whiteness) T = 0 min	0.56 (*p* = 0.028)	0.37 (NS)
L (whiteness) T = 30 min	1.28 (*p* = 0.012)	−0.29 (NS)
L (whiteness) T = 60 min	0.82 (*p* = 0.063, NS)	−0.66 (NS)
b* (yellowness) T = 0 min	−0.64 (*p* < 0.001)	0.23 (NS)
b* (yellowness) T = 30 min	−1.02 (*p* < 0.001)	0.21 (NS)
b* (yellowness) T = 60 min	−0.96 (*p* < 0.001)	0.22 (NS)

Data show mean changes from baseline for n = 30 subjects per group. NS = not significant.

## Data Availability

Data are available upon reasonable request to the corresponding author.
